# Arthroscopic Rotator Cuff Repair With Collagen Patch Augmentation: An In Vivo Examination of Biologic and Biomechanical Patches

**DOI:** 10.7759/cureus.115677

**Published:** 2026-09-02

**Authors:** Caroline Sumner, Srinath Kamineni

**Affiliations:** 1 Orthopaedic Surgery and Sports Medicine, University of Kentucky College of Medicine, Lexington, USA; 2 Orthopaedic Surgery, Indiana University School of Medicine, Evansville, USA; 3 Orthopaedic Surgery, Wabash General Hospital, Mount Carmel, USA

**Keywords:** biobrace, collagen patch augmentation, regeneten, revision arthroscopy, rotator cuff repair

## Abstract

The use of patch augmentation in the setting of large-to-massive rotator cuff repairs and poor tissue quality may reinforce native tissue and add structural integrity, potentially decreasing retear rates. Many patches are currently marketed for this use, with research focusing primarily on easily observable surgical outcomes, including range of motion, pain, and functionality.

These case reports investigate the use of two such patches, Regeneten (Smith+Nephew, Andover, MA, USA) and BioBrace ( CONMED, New Haven, CT, USA), observed at subsequent revision surgeries; this in vivo observation provides insight into the cellular effects of these patches, namely, the extent of healing and tissue proliferation. In case 1, Patient A received a Regeneten patch during initial repair; upon revision nine months later, staple fragments were found floating in the joint space, with no evidence of the previously placed patch except for bone anchors. In case 2, Patient B received a BioBrace patch during initial repair; upon revision five months later, the patch was found to be intact and well-integrated, with evidence of cellular overgrowth and neovascularity. These two cases, each a single point-in-time evaluation of a prior rotator cuff repair with patch augmentation, raise questions regarding a potential difference in the bioinductive properties of the two patches. Further large-scale studies are warranted to investigate the different bioinductive qualities of the patches and their role in the cellular healing process, as well as their unique complication risks.

## Introduction

Symptomatic rotator cuff tears reduce quality of life and shoulder function and can result from trauma, chronic overuse, or age-related degeneration [1]. More than 300,000 rotator cuff repairs are performed each year in the United States, but retear rates remain as high as 39%-94% [2]. The goal of such procedures is to repair the injured tendon and stabilize the glenohumeral joint, restoring both function and reducing pain levels [1]. Reasons for rotator cuff failures include suture “pull-through” at the suture-tendon interface and compromised tissue quality [3]. 

Patch augmentation with surgical rotator cuff repairs is a recent promising advance in the field, with documented lower retear rates, as well as improvements in pain, function, and range of motion [4-8]. This technique utilizes a biological patch to reinforce native, compromised rotator cuff tissue, with the aim of providing additional structural integrity. The ability to immediately reinforce the tendon repair with biomechanically strong implants can be useful in settings of poor tissue quality and revision of large to massive rotator cuff tears [3]. In larger rotator cuff tears, patch grafts can help close the space between tendon and tuberosity [9]. Active biological augmentation may also help compensate for poor local tissue quality by encouraging the release of growth factors at the site, allowing collagen integration between the patch and host tissue [10].

Regeneten, manufactured by Smith+Nephew, Andover, MA, USA, is an implant composed of highly porous, purified type I collagen from a bovine Achilles tendon [2,4,10,11]. The collagen scaffold has no significant mechanical strength but promotes tendon thickness, while the porous quality supports the adhesion of regenerative cells [4,10]. The patch is gradually incorporated over six months [11]. At two years post-operation, the Regeneten implant has been correlated with improved functional outcomes and increased tendon thickness without reports of serious adverse effects [2]. 

The BioBrace, manufactured by CONMED, New Haven, CT, USA, is another type of patch, a type I collagen scaffold reinforced with poly-L-lactide (PLLA) microfilaments [12,13]. The collagen-PLLA matrix promotes immediate load sharing and increased structural integrity, with the added quality of bioactive tissue ingrowth and remodeling. The patch supplies mechanical strength at time zero and becomes fully resorbed and integrated over two years [3,12,13]. The combination of bioinduction and integration with mechanical strength encourages increased tendon thickness while simultaneously providing mechanical support [3,12,13]. 

We present two cases of rotator cuff tears previously repaired using collagen patch augmentation. Patient A received a Regeneten patch, while Patient B received a BioBrace patch. We conducted this study in compliance with the principles of the Declaration of Helsinki; the study protocol was approved by the Institutional Review Board of the University of Kentucky (No. 102277), and informed consent was waived.

## Case presentation

Case 1: biologic patch (Regeneten)

Patient History

Patient A is a 58-year-old female. She had a right proximal humerus malunion seven years prior and right arthroscopic greater tuberosity osteoplasty with rotator cuff repair by subacromial depression and biceps tenotomy three years prior. The patient presented with right shoulder pain and was diagnosed with a high-grade partial supraspinatus rotator cuff tear (grade 2 fat atrophy) by magnetic resonance imaging (MRI) scan (Figures 1A-1B). After supervised physical therapy without improvement, the patient opted to undergo a right arthroscopic rotator cuff repair with possible augmentation.

**Figure 1:**
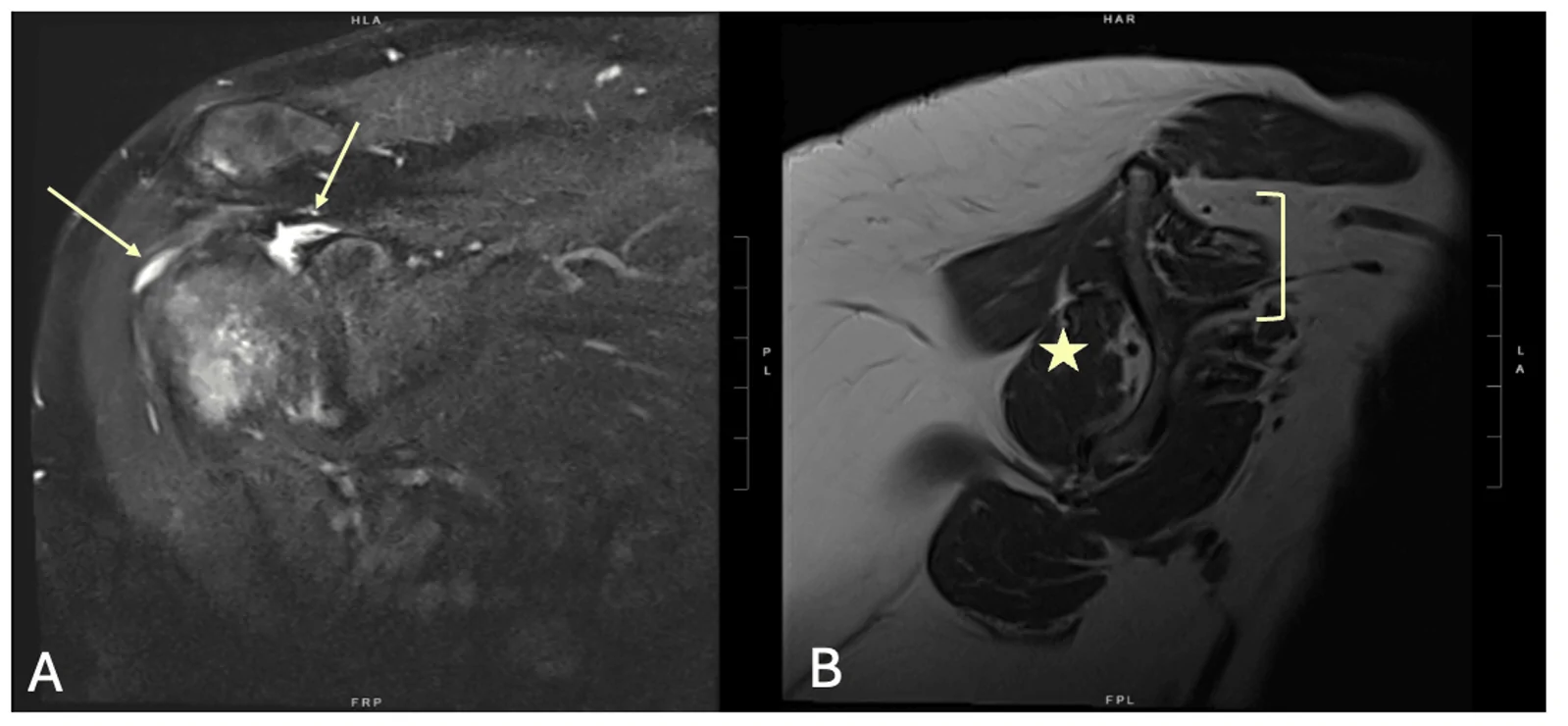
MRI of Patient A’s Initial Tear (A-B) Pre-operative MRI scan of Patient A’s right shoulder with the initial rotator cuff tear. T2-weighted coronal view (A) demonstrates hyperintense, fluid-filled defects (yellow arrows) of the supraspinatus where it inserts on the humeral head, with heterogeneity of the articular-sided fibers proximal to the footprint, indicating a tear. T1-weighted sagittal view (B) demonstrates grade 2 fat atrophy of the supraspinatus (yellow bracket) and subscapularis (yellow star), as indicated by hyperintense streaks of fat in the muscle and significant flattening of the supraspinatus muscle shape.

Initial Bioimplant Placement

At shoulder arthroscopy, the patient was found to have a complete tear, contrary to the MRI scan, involving the supraspinatus, with minimal retraction (Figure 2A) and adhesions that required release. Once the cuff was completely mobilized, a tension-free, intermediate row repair (Figure 2B) was possible with two double-loaded, all-suture anchors. This was followed by a patch graft augmentation. Two Regeneten patches were placed and held in position using two packs of tendon anchors and five bone anchors (Figure 2C). The patches were felt to be stable and under appropriate tension. There were no complications during the procedure. The patient had improved pain levels and range of motion at her early follow-up appointments.

**Figure 2:**
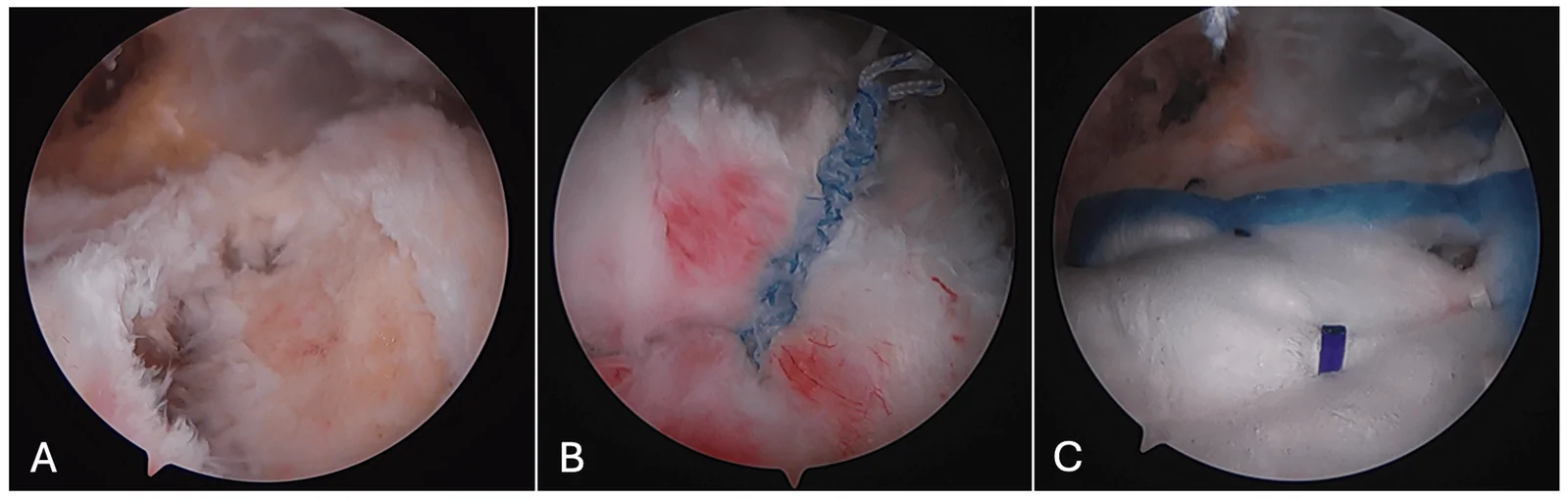
Regeneten Placement (A-C) Arthroscopic view of the Regeneten placement procedure in Patient A, showing snapshots of (A) the complete tear of the supraspinatus and partial tear of the infraspinatus, (B) the intermediate-row repair, and (C) placement of the two Regeneten patches.

Revision, Observations, and Outcome

At seven months post-procedure, the patient reported overall improvement but still had continued pain and had symptomatically been unable to perform formal physical therapy. Patient denied any instances of trauma or injury. Physical exam revealed weakness in forward elevation and abduction with a significant loss of above-shoulder-level active motion. Right shoulder X-rays revealed superior humeral head migration (Figure 3). Due to continued pain and weakness, functional limitations, inability to perform physical therapy, and imaging findings, the patient chose to proceed with a revision surgery. A revision arthroscopic rotator cuff repair was planned with Achilles tendon allograft superior capsular reconstruction (SCR), performed nine months after the original placement.

**Figure 3:**
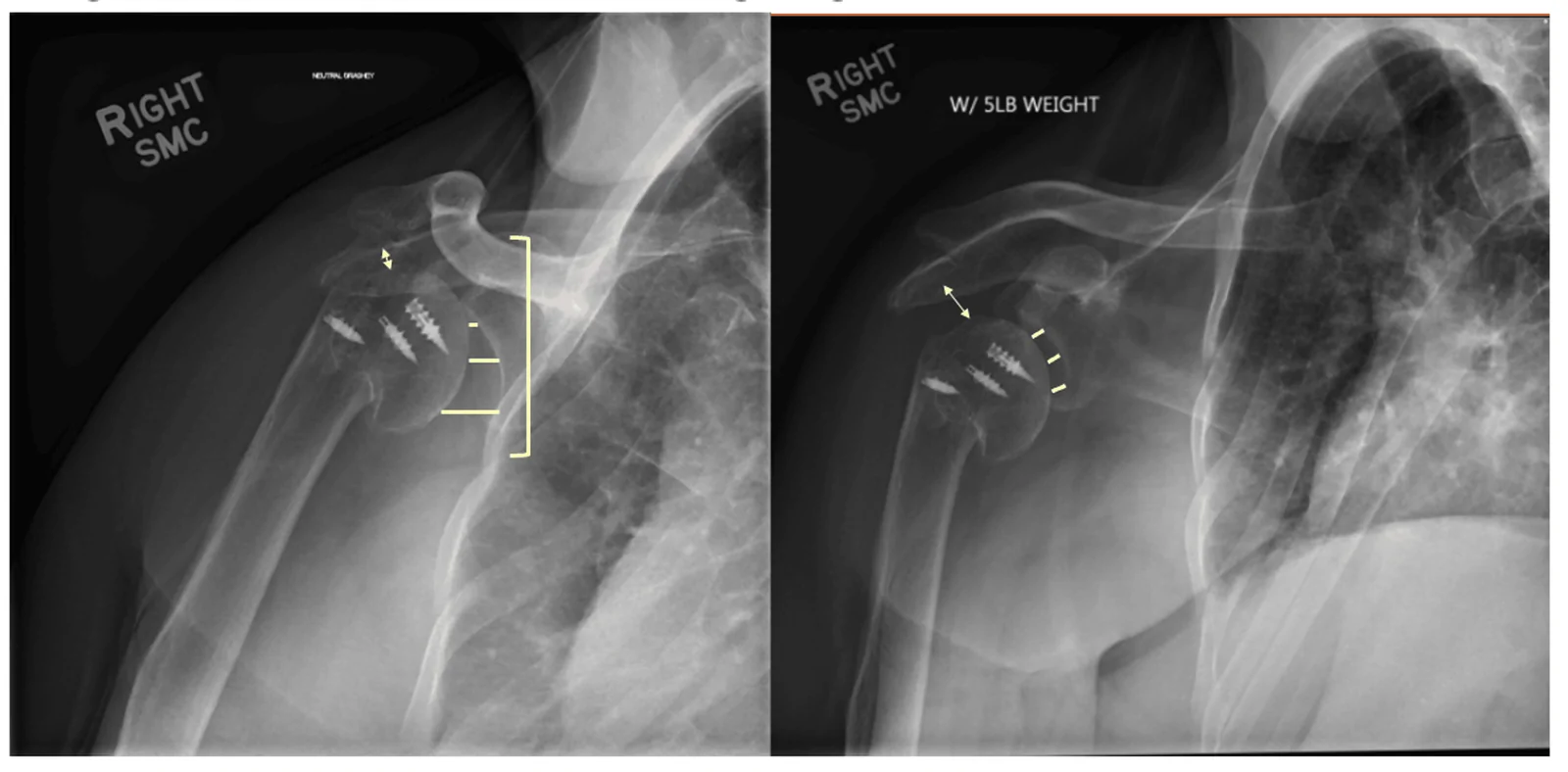
Shoulder Imaging Before Regeneten Revision Radiographic Grashey and AP weight-bearing (left to right) views of Patient A’s right shoulder before the revision. Superior humeral head migration is evident, indicating further tearing of the superior cuff (bracket). Narrowing of the glenohumeral joint and subluxation are demonstrated by the asymmetric space between the inferior and superior humeral head and glenoid (lines). The wider space between the inferior humeral head and glenoid indicates superior subluxation. There is narrowing of the subacromial space (double-pointed arrow). No abnormal subluxation of the humeral head is noted on weighted images (lines); dynamic superior migration is demonstrated by the reduction of migration with a 5-pound weight.

During the procedure, a well-aligned glenohumeral joint with moderate humeral head articular cartilage wear was observed. There was a complete full-thickness tear of the supraspinatus with retraction. There were adhesions and scarring, as well as a significant amount of synovitis, throughout the shoulder joint. Free-floating fragments of the previously used tendon fixation staples were evident throughout the joint, and bone anchors were still present in the greater tuberosity (Figures 4A-4B). There was no evidence of the previously placed patches. Debridement and removal of all staple remnants was performed, along with scar excision and cuff mobilization. The supraspinatus cuff remnant could not be approximated to the greater tuberosity, so an Achilles tendon allograft was deployed as an SCR with repair of the infraspinatus and subscapularis (Figure 4C). The graft was found to be stable and tensioned.

**Figure 4:**
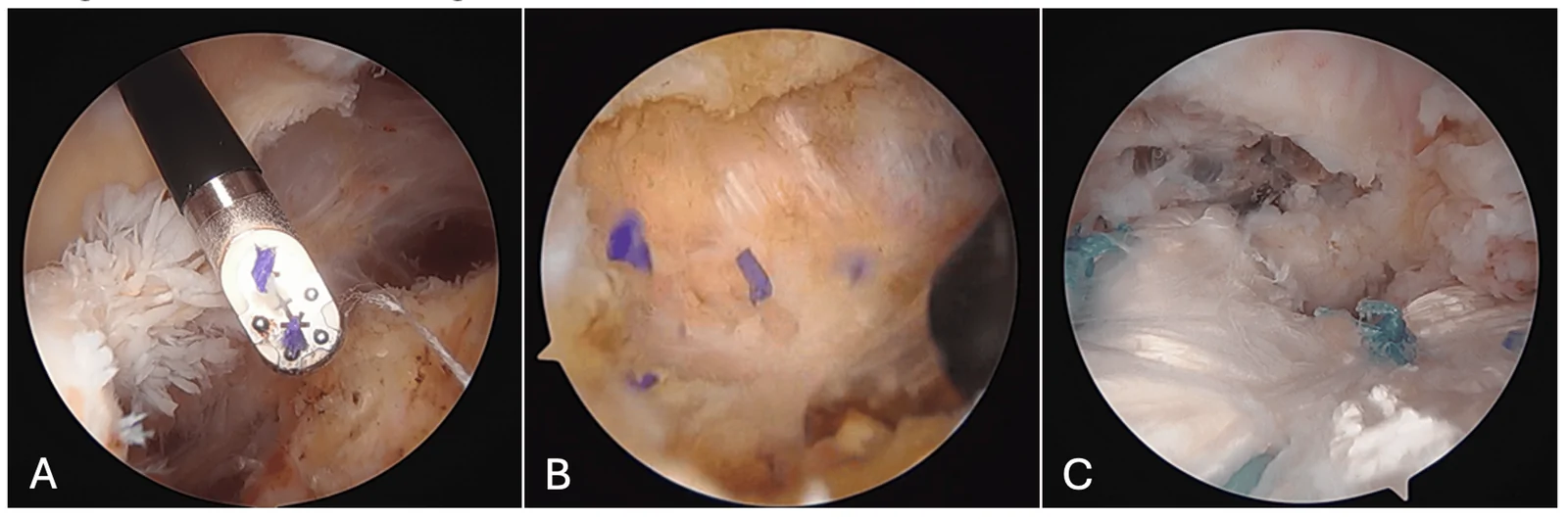
Regeneten Revision (A-C) Arthroscopic investigation of the patch during revision of Patient A. Fragments of the previously used purple tendon fixation staples were captured in the (A) shaver and (B) floating freely about the joint space, with no evidence of the previously placed Regeneten patch, other than the bone and soft-tissue anchor remnants. The revision was completed with an Achilles tendon allograft (C).

At the 11-week follow-up, the patient reported much-improved pain (1-2/10 VAS), though poor range of motion (30° active flexion, 30° active abduction, and 160° passive flexion). Radiographic imaging demonstrated decreased superior migration (Figure 5). The patient was lost to further follow-up.

**Figure 5:**
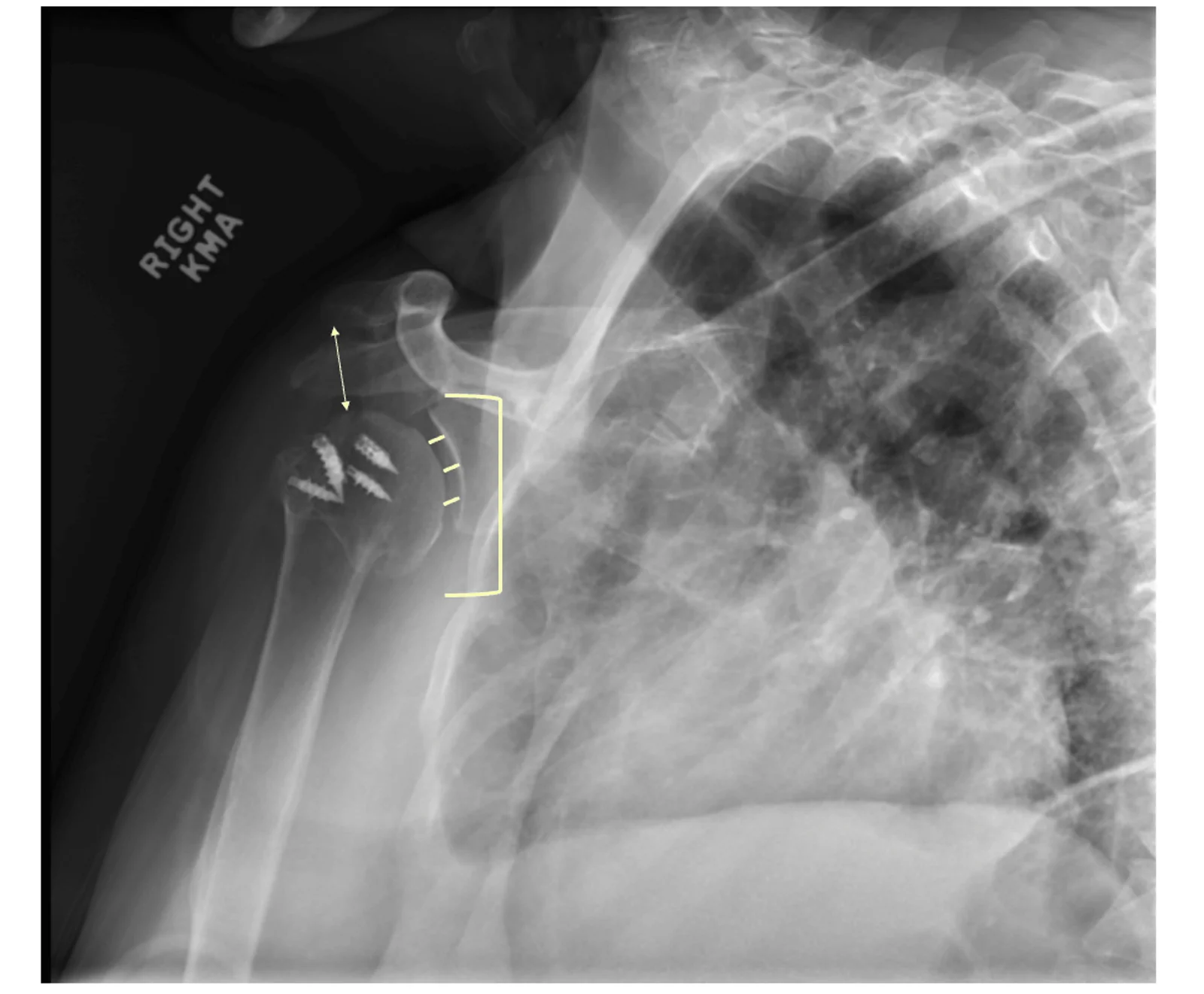
Case 1 Shoulder Imaging at 11-Week Revision Follow-Up Radiographic Grashey view of Patient A’s right shoulder at the 11-week follow-up appointment. Multiple screws are seen in the humeral head. Superior migration is greatly improved (double-pointed arrow) with concentric alignment at the glenohumeral joint (bracket), indicated by the symmetrical space between the humeral head and glenoid (lines).

Case 2: biomechanical patch (BioBrace)

Patient History

Patient B is a 36-year-old female with a long history of rotator cuff pain. One year prior, she had a traumatic fall and presented with significant left shoulder pain; conservative measures were attempted, but pain was recalcitrant to physical therapy and a corticosteroid injection. An MRI revealed traumatic tears of the rotator cuff and anterior labrum, so she opted to undergo an arthroscopic rotator cuff repair with labral repair. She reported lower levels of pain in early follow-ups and showed improved range of motion. After the operation, another traumatic fall and sharp pain following the lifting of a heavy animal caused her to return to the clinic complaining of significant worsening pain and decreased range of motion. MRI revealed a high-grade partial-thickness tear of the supraspinatus and anterior fibers of the infraspinatus (Figures 6A-6B). The patient opted to undergo left shoulder revision arthroscopic rotator cuff repair.

**Figure 6:**
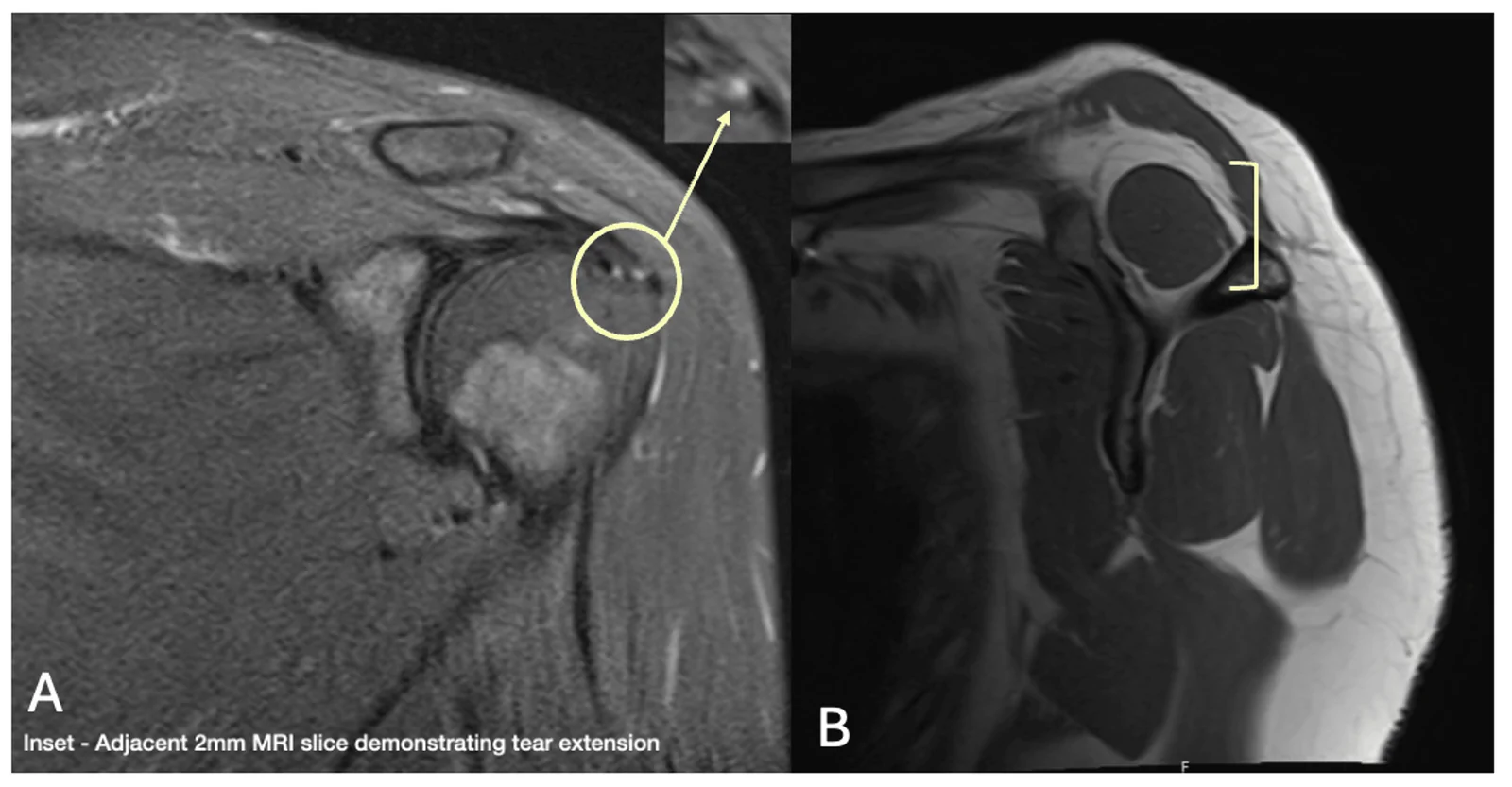
MRI of Patient B’s Initial Tear (A-B) Pre-operative MRI scan of Patient B’s left shoulder with the initial rotator cuff tear. T2-weighted coronal view (A) shows hyperintense fluid-filled defects of the supraspinatus footprint (circle); two signals merge into one in the adjacent slide (insert indicated by arrow), demonstrating an oblique tear extension. T1-weighted sagittal view (B) demonstrates possible grade 1 fat atrophy of the supraspinatus (yellow bracket), indicated by minor hyperintense fatty streaks and maintenance of its convex shape and muscle thickness.

Initial Bioimplant Placement

During the procedure, a well-aligned glenohumeral joint was observed with no significant cartilage wear, a long head of the biceps tendon in good condition, and an intact, previously repaired labrum. The patient had a high-grade bursal-sided, subtotal partial-thickness tear of the supraspinatus, extending from the rotator interval to the infraspinatus (Figure 7A). The tissue quality of the supraspinatus was noted to be friable and delaminated. The thin, joint-sided remainder of the cuff was taken down to complete the tear and debrided back to healthy tissue. An accessory lateral portal was created, and two double-loaded, 2.9-mm all-suture anchors were placed into the cuff margin of the greater tuberosity. These were passed through the medial cuff margin and tied to create a medial-row fixation (Figure 7B). The lateral portion of the supraspinatus was captured using the double-loaded Y-knot anchors about the greater tuberosity. The graft was draped over the rotator cuff tear, overlapping both medially and laterally, and the medial and lateral sutures were tied down to secure the graft (Figure 7C). The subscapularis and infraspinatus were secured to the graft anteriorly and posteriorly, respectively. The rotator cuff appeared stable and under appropriate tension. There were no complications during the procedure. 

**Figure 7:**
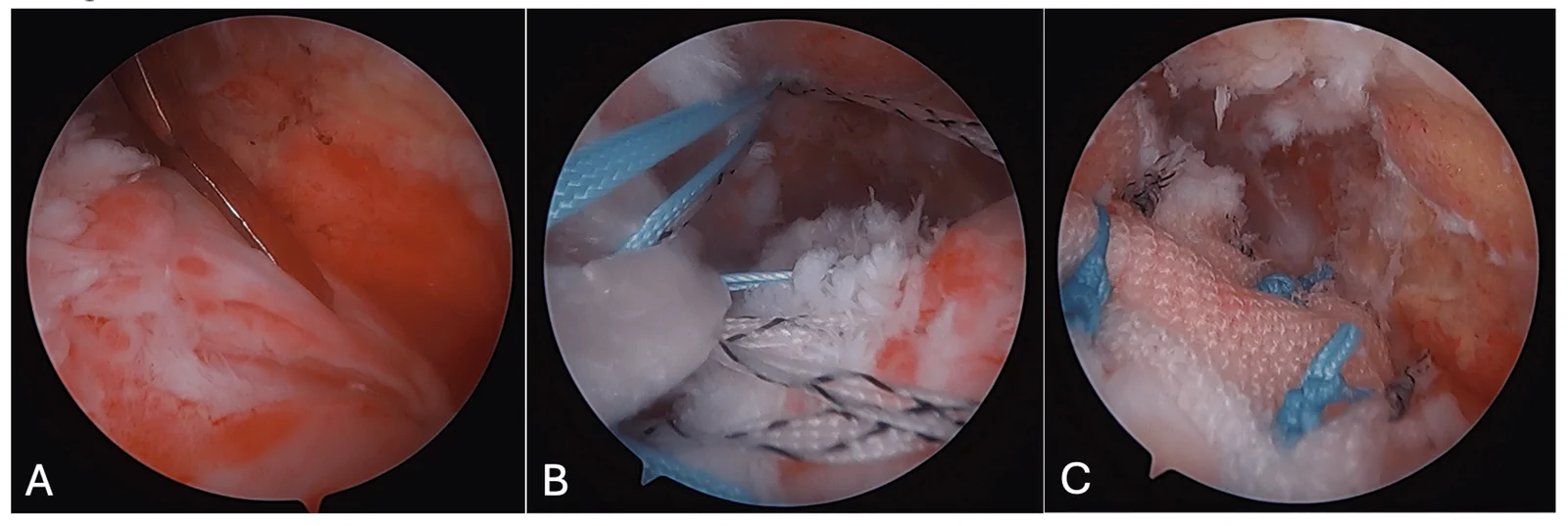
BioBrace Placement (A-C) Arthroscopic view of Patient B’s (A) partial-thickness tear of the poor-quality supraspinatus, (B) repaired with a medial row fixation. (C) A BioBrace was used to augment the repair.

The patient underwent physical therapy and progressed until two months post-surgery when she struggled with sudden increased pain, with multiple falls retrospectively documented. Radiographic images were taken and revealed no signs of humeral head migration (Figure 8). She attempted conservative management for another month and achieved very limited active painful motion and functionality at her three-month follow-up. Because she has a pacemaker, it had proven difficult to obtain an MRI. In the setting of new traumatic injury with continued pain, limited range of motion, and decreased functionality, she chose to proceed with a diagnostic arthroscopy. 

**Figure 8:**
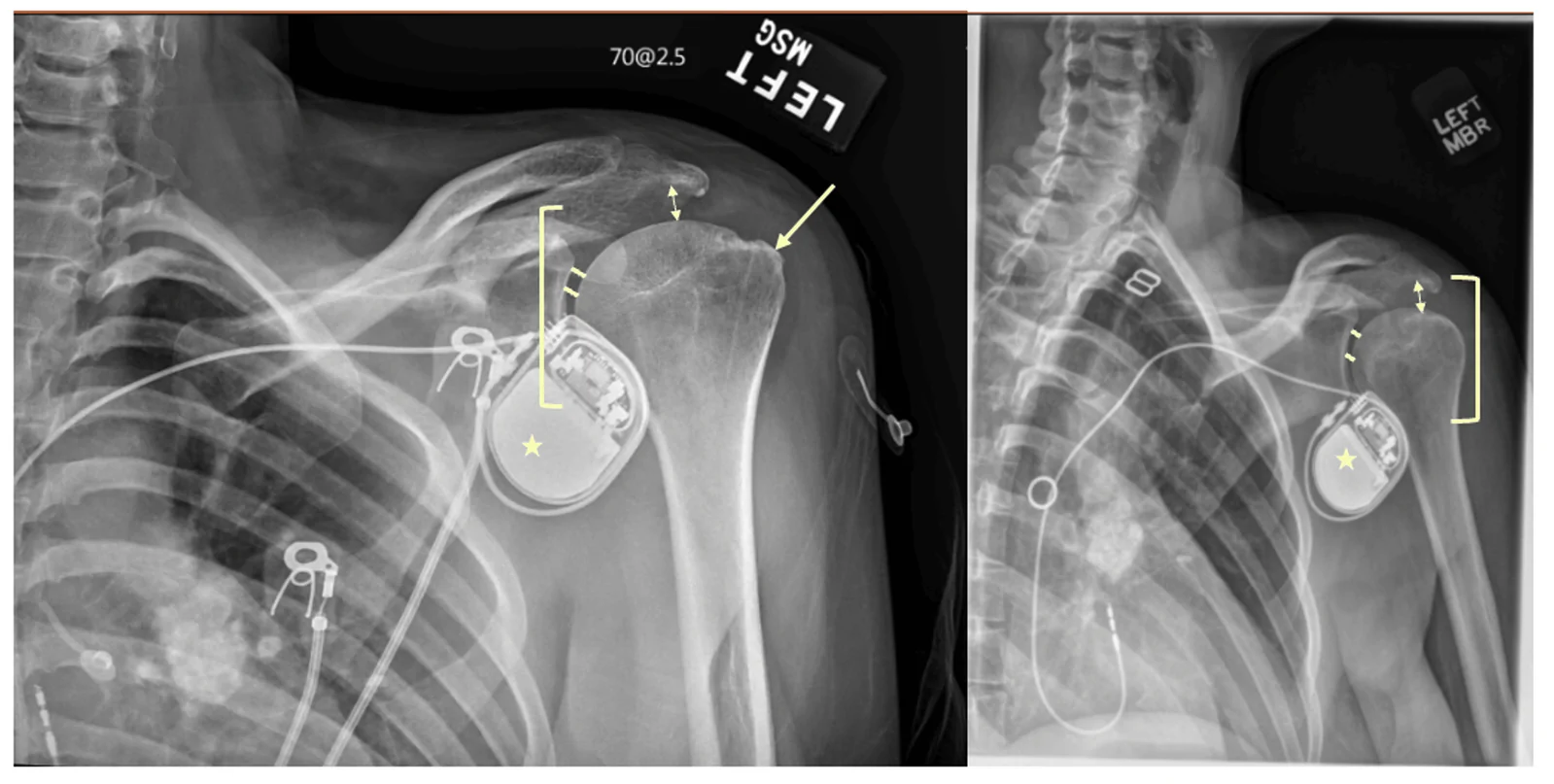
Shoulder Imaging Before BioBrace Revision Radiographic AP and Grashey (left to right) views of Patient B’s left shoulder before the revision. The AC and glenohumeral joints are normally aligned (bracket, lines). Migration was not an issue in this patient, as evidenced by the normal subacromial space (double-pointed arrow). There is contour irregularity and sclerosis of the greater tuberosity (single-pointed arrow) in the AP view, which is indicative of a chronic tear. Pacemaker artifact is present (star).

Revision, Observations, and Outcome

Five months after her rotator cuff repair with BioBrace augmentation, Patient B underwent an arthroscopic revision. The patient was found to have an intact and biologically integrated previous supraspinatus repair and BioBrace augmentation. The previous repair was extensively probed on the joint and bursal sides, without signs of adverse reaction, tearing, or delamination. The cuff was well healed onto the tuberosity, with the patch showing noticeable integration and neovascularity (Figures 9A-9B). Cellular overgrowth with the presence of new vessels demonstrates both the integration of the patch into native tissue and growth of new tissue. The infraspinatus at the posterior interface of the BioBrace, the edge of the previous repair, was torn and delaminated (Figure 9C). This new tear was debrided and repaired with a second BioBrace augmentation (Figure 9D).

**Figure 9:**
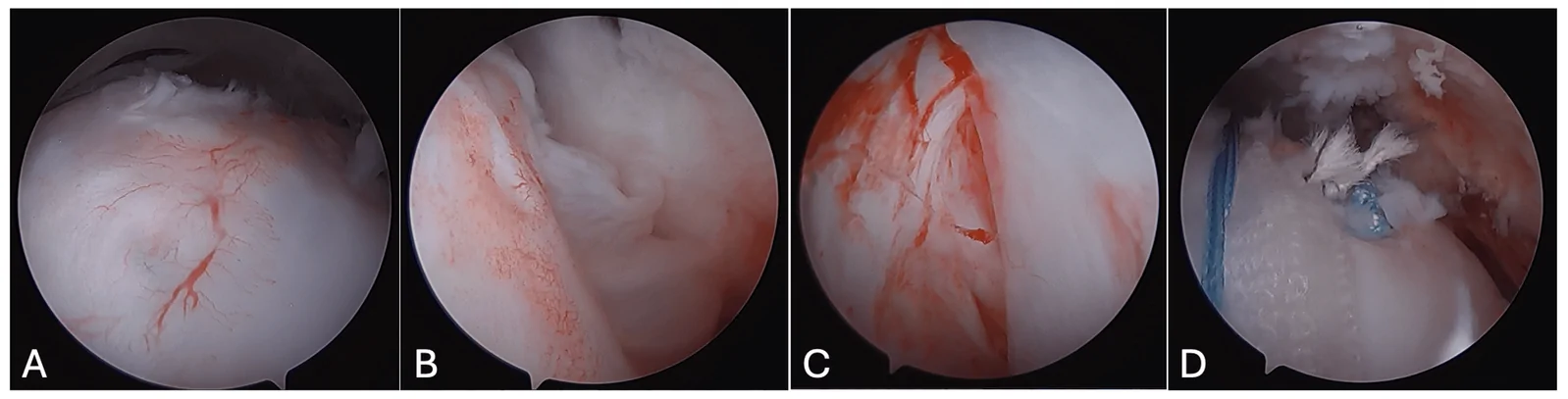
BioBrace Revision (A-D) Patient B’s previous repair was fully intact with no signs of tearing or delamination. The patch was well-healed onto the tuberosity and showed signs of overgrowth and neovascularization (A, B), with collagen and small vessels showing through the new tissue. The tear occurred at the interface between the previous patch and the infraspinatus (C) and was repaired with another BioBrace (D).

MRI at the seven-week follow-up showed the supraspinatus and infraspinatus to be well healed and well-integrated, with a significantly thickened supraspinatus tendon with good interface at the tuberosity (Figure 10A). At three-month follow-up, the patient reported improved pain levels (1-3/10 VAS) and had improved passive range of motion (160° flexion, 150° abduction, and 50° external rotation). At 12-month follow-up, the patient reported increased pain due to several falls, though radiographic imaging showed normal joint alignment (Figure 10B). After 15 months, she had another traumatic fall that required an arthroscopic biceps tenodesis and re-anchoring of the prior augmentation using FiberWire® (Arthrex, Inc., Naples, FL, USA); 13 months after this final procedure, she had improved active range of motion (170° flexion and 160° abduction), though her pain level had increased (6/10 VAS).

**Figure 10:**
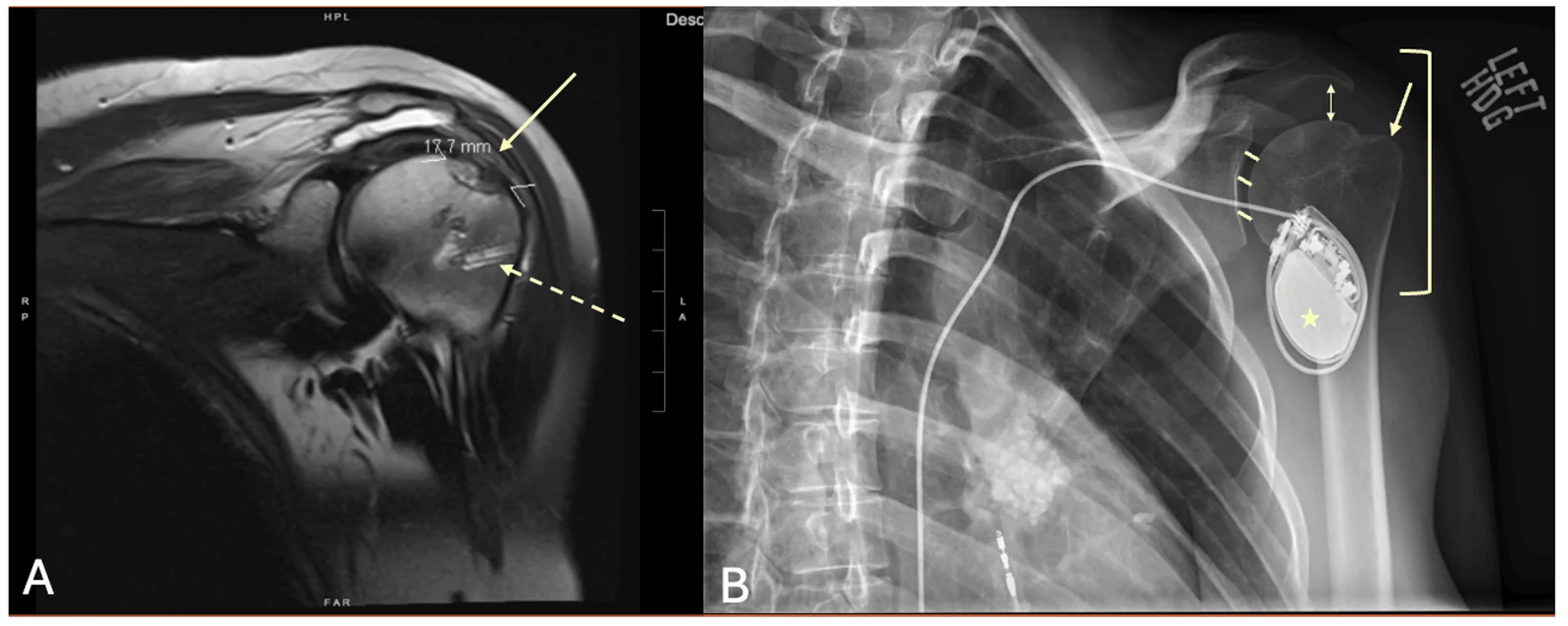
Case 2 Shoulder Imaging at Revision Follow-Up (A-B) MRI scan (A) of Patient B’s left shoulder seven weeks after the revision. Both the supraspinatus and infraspinatus appear to be well-healed and well-integrated, with a significantly thickened supraspinatus tendon and good interface at the tuberosity (single-pointed arrow). A lateral fixation all-suture anchor is seen within the humeral head (dashed arrow). Radiographic Grashey (B) view of the shoulder at the 12-month follow-up shows normal AC and glenohumeral joint space alignment (double-pointed arrow, bracket, lines). The greater tuberosity has a more regular contour (single-pointed arrow) compared to the preoperative image. Pacemaker artifact is present (star).

## Discussion

The use of patch augmentation in arthroscopic repair of large-to-massive and revision rotator cuff tears has grown in popularity. The potential decrease in retear risk would improve patient outcomes, including pain, range of motion, and functionality, and decrease the need for further operative management. Much of the literature has focused on the initial placement and subsequent postoperative measures. We had the opportunity to re-examine the bioinductive implants in vivo on two occasions, observing levels of macroscopic patch integrity and integration, as well as the state of the cuff repairs. Upon in vivo inspection, the Regeneten had not integrated, with fragments found floating in the joint space. The BioBrace patch was found to be well-integrated, with evidence of new cellular and vascular growth.

The biologic patch used here, Regeneten, is made of bovine collagen and is designed to enhance the healing response and thicken the augmented tendon. Some studies have shown that using the bioinductive implant improves clinical outcomes and significantly reduces retear rates [4,14]. One randomized trial found that six-month postoperative MRIs of Regeneten implants showed significantly increased tendon thickness and integration with the native tendon compared to those who received a typical sutured repair [15]. Other studies, however, reported that the use of a bioinductive collagen implant was not associated with reduced retear rates and was associated with increased rates of postoperative stiffness [16,17].

The Regeneten patch, implanted in Patient A, demonstrated potential degradation, with loose and free-floating staples. During the initial arthroscopic rotator cuff repair, the Regeneten patch was intended to augment the cuff tear. Nine months later, at revision surgery, no remnants of the patch were found. This suggests that the patch had either degraded, been reabsorbed too soon, or migrated. Many fragments of bone and soft-tissue anchors were found floating freely throughout the joint space, posing a tribology risk. There was no evidence of any patch remaining, but rather a complete full-thickness tear of the previously repaired supraspinatus with retraction. The surgical team performed the revision rotator cuff repair using a different augmentation method.

The biomechanical patch used, BioBrace, can provide both mechanical strength and encourage growth and healing of native tissue due to its highly porous type I collagen scaffold reinforced with PLLA microfilaments [18]. The combination of strength and biological properties is of great importance in large-to-massive and revision rotator cuff repairs [19]. Previous literature has demonstrated its structural strength in sheep models and its utility in rotator cuff augmentation [3,19,20]; however, there have been few studies reporting on postoperative measures. There are some concerns regarding the patch’s high cost [11] and the potential for local host immune responses to the PLLA microfilaments, though this response was minimal in sheep models [20].

The BioBrace patch used in the repair of Patient B’s rotator cuff was intact, with noticeable overgrowth and visible neovascularity. The BioBrace was initially used to reinforce a high-grade partial-thickness tear of a poor-quality supraspinatus. Five months later, revision arthroscopy was required, and the patch was observed. Where the BioBrace was previously placed on the cuff, new tissue had formed; bits of the BioBrace showed through from underneath the collagen overgrowth, indicating collagen incorporation of the patch. Blood flow and blood vessels were also observed in the area of BioBrace placement, demonstrating neovascularity. Both the supraspinatus and previous patch augmentation were in great condition, with only minimal fraying at the superior edge of the patch. No remnants were found in the joint space. There were no signs of patch failure or supraspinatus tears, and the cuff appeared to be well healed at the site of augmentation. Posterior to the edges of the BioBrace, the patient was noted to have delamination and tearing of the infraspinatus. A dramatic change in modulus between the normal muscle and the less-elastic, scarred neo-tendon creates a vulnerable space. The tear at the interface of the patch and cuff, an area not reinforced by augmentation, could raise concern for a phenomenon similar to that of a stress riser: the presence of a reinforced structure next to a vulnerable structure increases the risk of damage. The surgical team performed the revision rotator cuff repair with the addition of a second BioBrace. 

During the revision procedures for both cases, macroscopic examination of the prior patch augmentations offered insight into the patches’ biologic properties and healing processes, rather than relying on MRI signals alone. In this singular case, the Regeneten patch repair failed, and significant debris was found floating in the joint space, with no signs of neovascularization, posing a tribology risk. This is not consistent with the literature and instead reveals a lack of integration and tendon thickening after nine months. BioBrace, on the other hand, was well-integrated into the native tissue, with signs of neovascularization and healing after only five months in this specific patient. The tear discovered in the BioBrace revision, however, raises concern for a different complication: a tear adjacent to the patch interface due to the difference in tissue strength. There were no signs of harm from an over-robust immune response to the patch’s microfilaments, consistent with the literature focused on animal models. The difference in observations of these two patches - complete absence versus robust integration - may be a result of their fundamental design. Regeneten, designed purely to stimulate healing rather than provide immediate mechanical strength, may have reabsorbed too quickly in this high-stress revision case. The reinforced BioBrace scaffold likely provided additional mechanical support to preserve the collagen matrix during host cell infiltration and integration.

Due to differences between the cases and the inclusion of only two patients, no direct comparison can be made. The examination of a previously placed collagen patch is made possible only by the need for revision, inherently limiting the number of patients and generalizability of results. These two patients are different ages, with different mechanisms of injury, tissue quality, and surgical histories. The patients underwent revision at different time points in the healing process (nine vs. five months), and physical therapy adherence, risk of trauma, and daily activity can never be entirely controlled. Although this is a point-in-time examination of the state of the collagen patches at revision, the limited follow-up of Patient A is a weakness of this case report.

## Conclusions

These two cases granted the unique opportunity to examine two bioimplants in vivo during revision arthroscopy, demonstrating the need for further research. In the first case, the Regeneten patch was not integrated, and fragments were found floating throughout the glenohumeral joint. In the second case, the BioBrace was intact, and the cuff showed signs of new vascular and cellular growth. During the revision operations, the surgical team decided to use another BioBrace for Patient B but opted to pivot and use a different form of patch augmentation for revision in Patient A. These independent cases highlight a need for comparative and large-scale studies to determine differences between the two patches, including their biologic effects, ideal applications, and unique complication risks.
